# Wafer-scale and selective-area growth of high-quality hexagonal boron nitride on Ni(111) by metal-organic chemical vapor deposition

**DOI:** 10.1038/s41598-019-42236-4

**Published:** 2019-04-05

**Authors:** Hokyeong Jeong, Dong Yeong Kim, Jaewon Kim, Seokho Moon, Nam Han, Seung Hee Lee, Odongo Francis Ngome Okello, Kyung Song, Si-Young Choi, Jong Kyu Kim

**Affiliations:** 1 Department of Materials Science and Engineering, Pohang University of Science and Technology (POSTECH), Pohang, 37673 Republic of Korea; 2 Materials Modeling and Characterization Department, Korea Institute of Materials Science (KIMS), Changwon, 51508 Republic of Korea

## Abstract

We demonstrate wafer-scale growth of high-quality hexagonal boron nitride (h-BN) film on Ni(111) template using metal-organic chemical vapor deposition (MOCVD). Compared with inert sapphire substrate, the catalytic Ni(111) template facilitates a fast growth of high-quality h-BN film at the relatively low temperature of 1000 °C. Wafer-scale growth of a high-quality h-BN film with Raman E_2g_ peak full width at half maximum (FWHM) of 18~24 cm^−1^ is achieved, which is to the extent of our knowledge the best reported for MOCVD. Systematic investigation of the microstructural and chemical characteristics of the MOCVD-grown h-BN films reveals a substantial difference in catalytic capability between the Ni(111) and sapphire surfaces that enables the selective-area growth of h-BN at pre-defined locations over a whole 2-inch wafer. These achievement and findings have advanced our understanding of the growth mechanism of h-BN by MOCVD and will contribute an important step toward scalable and controllable production of high-quality h-BN films for practical integrated two-dimensional materials-based systems and devices.

## Introduction

Hexagonal boron nitride (h-BN) is a two-dimensional (2D) layered material which is an insulating isomorph of graphene with alternating boron and nitrogen atoms^1–3^. With outstanding physical properties and chemical stability^4–6^, h-BN has emerged as a key component in a variety of applications such as an ideal dielectric or substrate layer for graphene devices^7,8^, tunneling barrier^9^, and deep-ultraviolet emitter^10,11^. In order to realize such promising applications of h-BN, remarkable progress in material synthesis is required: such as the scalable growth of uniform high-quality h-BN and the precise site-control of nucleation sites based on a deep understanding of its growth mechanism.

Chemical vapor deposition (CVD) on catalytic transition metal substrates such as Ni^12,13^, Cu^4,14^, Pt^15^ has been widely adopted for the growth of h-BN layers. However, conventional CVD systems typically hold a horizontal tube furnace with a limited diameter, so that the scale-up of h-BN synthesis is significantly restricted. Alternatively, metal-organic chemical vapor deposition (MOCVD), a well-established technique for industrial-scale growth of III-nitride epitaxial films, has been proposed as a promising method to attain wafer-scale h-BN films on sapphire or other substrates. Y. Kobayashi *et al*. demonstrated MOCVD-grown h-BN layers on sapphire, utilizing it as a releasing layer for transferring GaN-based optoelectronic devices^16,17^. Q. S. Paduano *et al*. investigated the effects of temperature, pressure, and V/III ratio on the MOCVD growth of h-BN on sapphire and its self-terminating behavior^18,19^. Recently, wafer-scale uniform growth of h-BN films on sapphire was reported, in which the effects of reactor pressure and types of carrier gas were investigated^20,21^. However, MOCVD-grown h-BN films on inert sapphire substrates have not progressed to a reliable quality for practical applications because extremely high temperatures greater than 1400 °C are typically required to obtain a high-quality film^22–24^. This increases the inhomogeneity of the surface temperature over inert substrates, and damages SiC and quartz parts in MOCVD reactors, thus, posing a significant limitation in productivity and cost effectiveness. On the other hand, transition metal substrates such as Ni^25^ can facilitate the growth of h-BN at relatively low temperatures while maintaining the advantages offered by MOCVD including multi-wafer scale growth and very precise control over the combination of complex growth parameters. However, there has been little study on the wafer-scale growth of h-BN layers on metal substrates using MOCVD. In addition, the understanding and utilization of the catalytic effect of transition metal substrates during MOCVD growth can enable a site-controlled, i.e., selective-area growth of h-BN that can realize a fundamental micro-scale “h-BN building block” for integrated 2D materials-based devices and systems. Compared with many attempts for scale-up synthesis and spatially-controlled growth of other 2D materials including graphene^26,27^, little efforts have been made for a large-and-selective area growth of high-quality h-BN by MOCVD.

In this study, we report the wafer-scale growth of high-quality few-layer h-BN film on Ni(111) template at the relatively low temperature of 1000 °C using a commercial multi-wafer MOCVD reactor. Various characterization tools including Raman spectroscopy, high-resolution transmission electron microscopy (HR-TEM), near-edge X-ray absorption fine structure (NEXAFS) spectroscopy, and X-ray photoelectron spectroscopy (XPS) were used to compare the microstructural and chemical characteristics of the MOCVD-grown h-BN films on the Ni(111) template and sapphire substrates. It was suggested that the adsorption and decomposition of NH_3_ and its radicals is catalytically facilitated on the Ni(111) surface, whereas the NH_3_ precursor is incompletely decomposed on the sapphire, resulting in a substantial difference in the growth kinetics and quality of the h-BN films. Based on the experimental results, we propose a promising route for the micro-scale selective-area growth of h-BN at desired locations over a whole 2-inch wafer using conventional lithography techniques. We believe this can be a significant progress toward the multi-wafer scale production of high-quality h-BN building blocks for integrated 2D materials-based devices and systems.

## Results and Discussion

**Figure 1:**
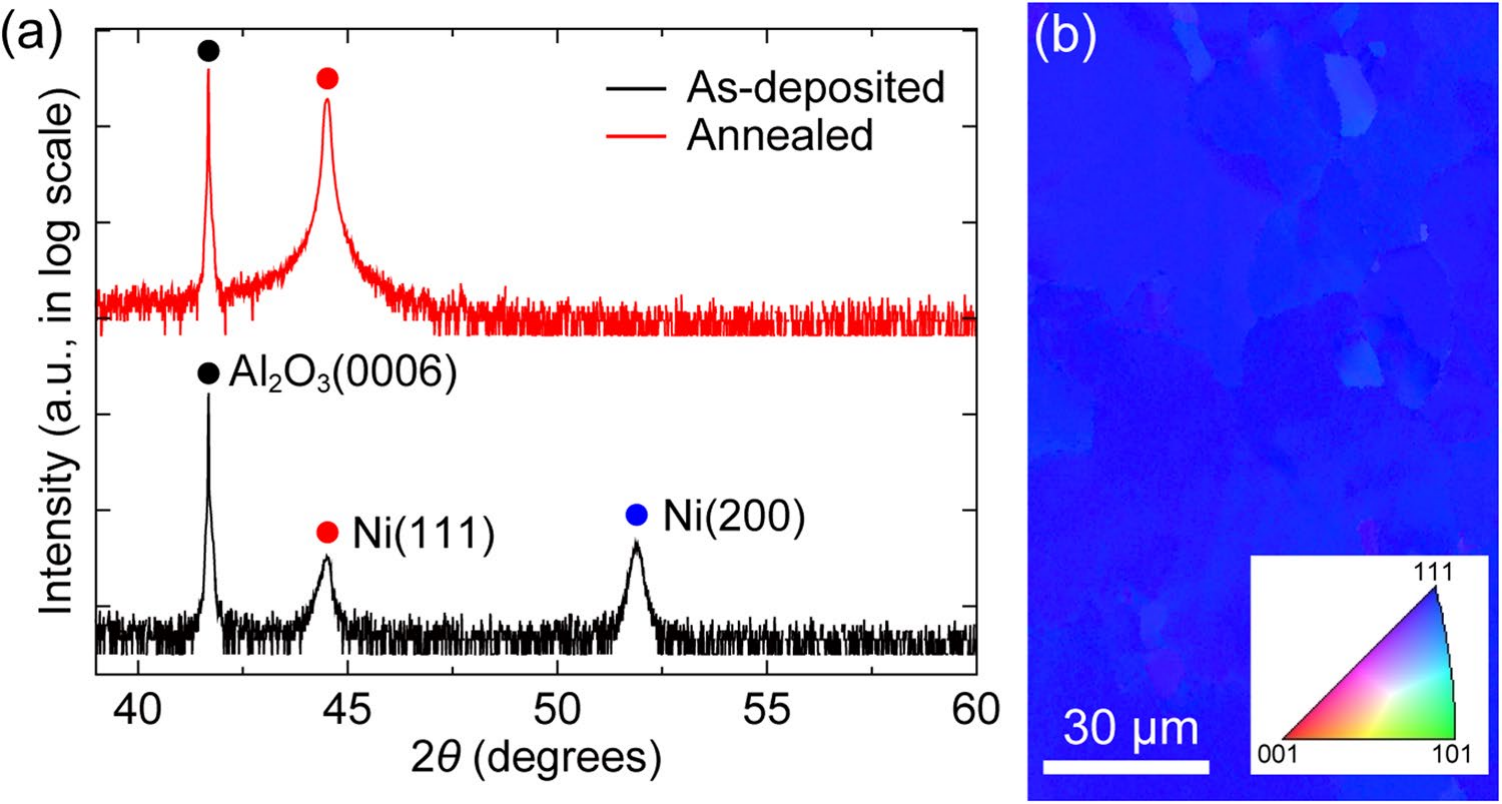
Crystallographic characterizations of the Ni(111) template. (**a**) *θ*−2*θ* XRD profiles of the Ni template before and after the thermal annealing. (**b**) EBSD map of the annealed Ni(111) template. (Inset) Inverse pole figure color triangle for crystallographic orientations.

The growth of h-BN on a Ni(111) template on sapphire was carried out using a commercial multi-wafer MOCVD system. Prior to the growth, the Ni(111) template was prepared by depositing a 600 nm thick Ni thin film on a 2-inch *c*-plane sapphire substrate by using a sputtering system. Then, the as-deposited Ni template was annealed in the MOCVD reactor at 1050 °C and 150 mbar for 20 min under H_2_ ambient to improve surface flatness and promote the growth of grains with (111) crystallographic orientation in the Ni film^28^. In fcc metals, the surface/interface energy of (111) planes is the lowest among all crystallographic orientations^29^, therefore, the growth of Ni grains with (111) preferred orientation becomes dominant to minimize the energy between Ni and underlying substrate. The crystallinity and surface quality of the Ni(111) template obtains an *epi-ready* condition after the thermal annealing as revealed by X-ray diffraction (XRD) patterns, electron backscatter diffraction (EBSD) image (Fig. 1), scanning electron microscopy (SEM) images, and atomic force microscopy (AFM) (Supplementary Fig. S7). As shown in Fig. 1a, the Ni (111) peak becomes larger and sharper while the Ni (200) peak disappears after the annealing, indicating the growth of Ni grains with (111) preferred orientation. The EBSD map of the Ni(111) template after the annealing clearly shows that the individual grains have a uniform (111) orientation with the average grain size of approximately 75 μm (Fig. 1b). The surface of the Ni(111) template after the annealing is very smooth with a root-mean-square (RMS) roughness of 0.605 nm, while the as-deposited Ni template has a much rougher surface topography (see Supplementary Fig. S7).

**Figure 2:**
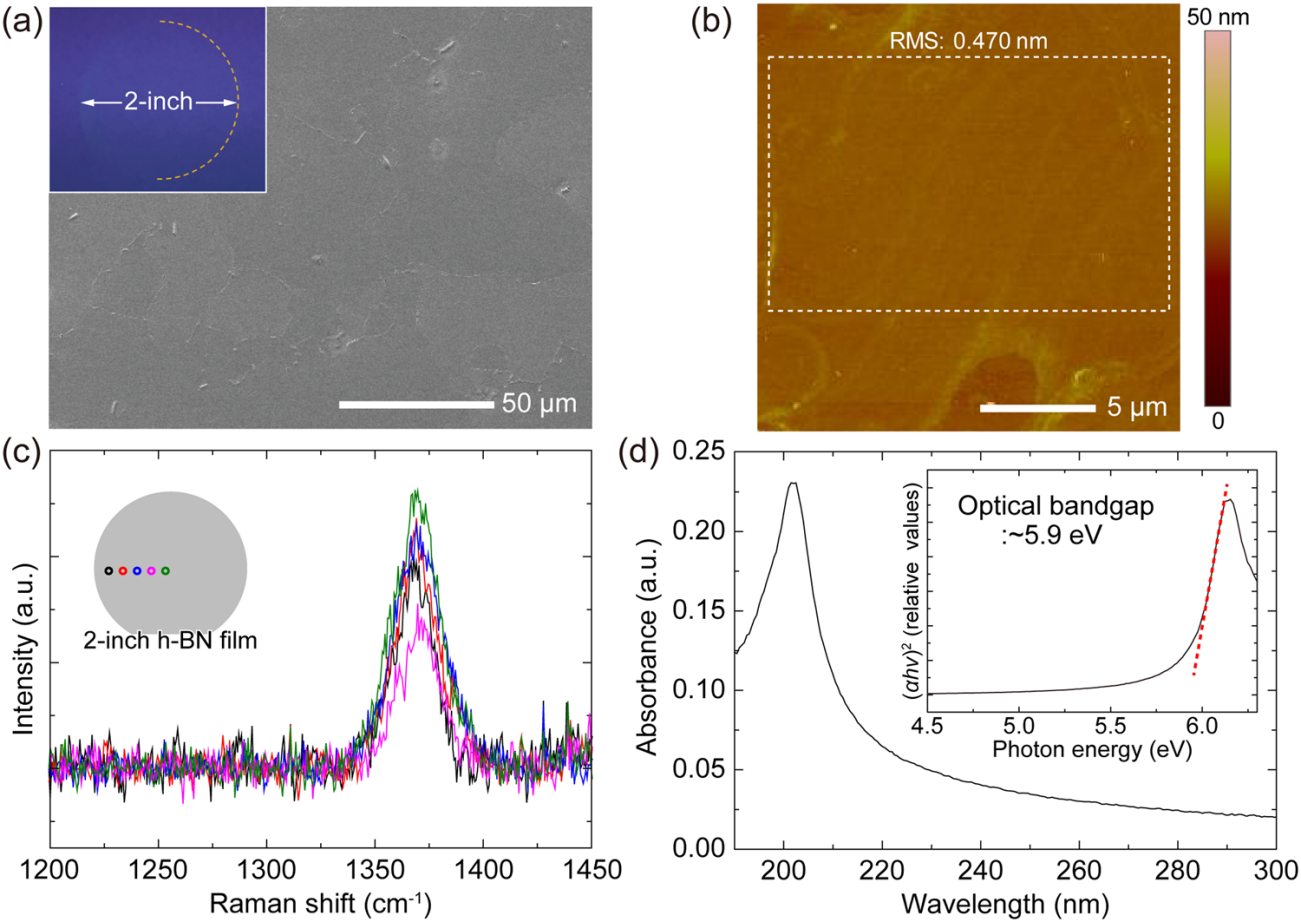
Microscopic and spectroscopic characterizations of the h-BN film grown on the Ni(111) template. (**a**) SEM image of as-grown h-BN film on the Ni(111). (Inset) Photograph of the 2-inch wafer-scale h-BN film transferred on a 4-inch SiO_2_/Si substrate. (**b**) AFM image of the h-BN film transferred on a SiO_2_/Si substrate. (**c**) Raman spectra of the h-BN film. (Inset) Schematic description of the representative positions where the Raman spectra were measured. (**d**) Absorbance spectrum of the h-BN film. (Inset) Tauc’s plot and optical band gap analysis.

After the thermal annealing, a h-BN film was grown at 1000 °C and 30 mbar for 50 pulsed injection periods of triethylborane (TEB) and NH_3_ as B and N precursors, respectively. The pulsed-mode growth whereby TEB and NH_3_ are alternatively injected into the reactor with an interruption time was applied to suppress parasitic reactions between B and N gas-phase precursors^30,31^. Figure 2a is a top-view SEM image of a part of the as-grown h-BN film on the Ni(111) template. The h-BN film on the Ni(111) exhibits a flat surface, similar with the 1050 °C-annealed Ni(111) template before the h-BN growth, while the irregular formation of thicker triangular h-BN domains were observed on the defective regions along the grain boundaries. Note that the defective features on the surface are discussed in Supplementary Note 1. The uniform SEM contrast over the whole 2-inch wafer area indicates a uniform h-BN film may be continuously grown over the entire wafer area. The inset of Fig. 2a demonstrates 2-inch wafer-scale growth and transfer of a h-BN film. The surface morphology of the h-BN film transferred on a SiO_2_/Si substrate was measured using AFM. Figure 2b shows the flat region within the grain, revealing a sub-nanometer smooth surface with a RMS roughness of 0.470 nm (see additional AFM image in Supplementary Fig. S8). In addition, no wrinkle was observed on the whole surface through SEM and AFM measurements. The wrinkle-free feature may result from the strong interfacial interaction between h-BN and Ni(111)^32,33^. To confirm the uniform existence of the h-BN film and its quality, spectroscopic characterizations were carried out. Figure 2c shows the Raman spectra measured at the five positions as depicted in the inset of Fig. 2c. There are distinct peaks with similar intensities at about 1370 cm^−1^, which correspond to the E_2g_ vibration mode of h-BN^34,35^, observed at all the positions over the 2-inch wafer area. The full width at half maximum (FWHM) values of the E_2g_ peaks are approximately 18~24 cm^−1^, comparable to those of monolayer h-BN films exfoliated from sintered crystals^35^, indicating a high-quality h-BN is grown on the Ni(111). Optical absorption spectroscopy was performed for the h-BN film after transfer onto a double-side polished sapphire substrate. The absorbance spectrum exhibits a significant peak at around 202 nm. As shown in the inset of Fig. 2d, the optical bandgap (*E*_g_) derived from the spectrum is approximately 5.9 eV in good agreement with the previously reported values^12,36^. The material characterization results demonstrate that a high-quality, wafer-scale h-BN film can be grown on a Ni(111) template at the relatively low growth temperature of 1000 °C by using a MOCVD system. It is a distinguishable result from previously reported h-BN growth on sapphire in which harsh growth conditions (>1400 °C) were required owing to the lack of catalytic reaction pathways^22–24^. To investigate the catalytic effect of the substrate on MOCVD growth of h-BN films, we compared the difference between h-BN growth on the Ni(111) and sapphire. On bare sapphire substrate, h-BN film was grown under identical growth conditions except for the higher growth temperature of 1050 °C and prolonged growth time of 200 source injection periods owing to much slower growth rate on sapphire than on the Ni(111) (see Supplementary Note 2). We confirmed the growth of h-BN on sapphire by measuring Raman and optical absorption spectroscopy (Supplementary Fig. S9). However, the FWHM value of the Raman peak is 43.2 cm^−1^, much larger than those of h-BN on the Ni(111), indicating much lower crystallinity of the h-BN film on sapphire. The remarkable differences in both growth rate and material quality indicate that the catalytic effect of the substrate is a critical factor for MOCVD growth of h-BN.

**Figure 3:**
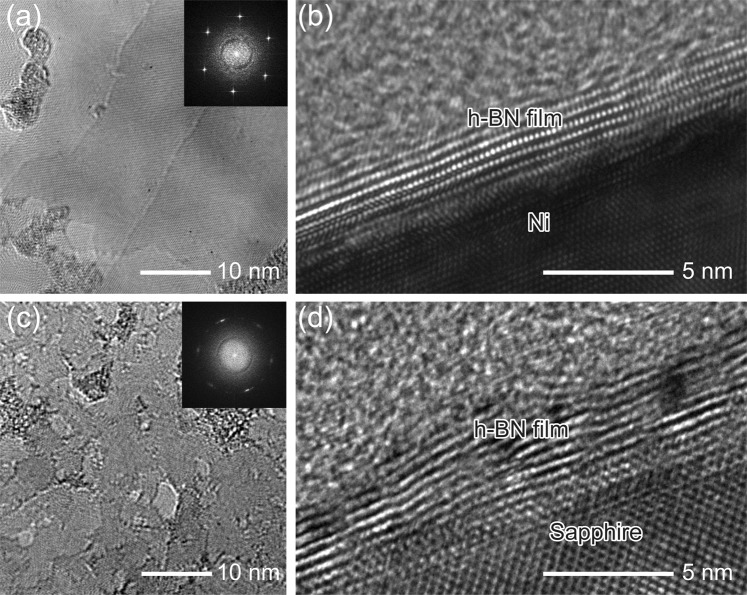
HR-TEM investigations. Plan-view HR-TEM images of the h-BN films grown on (**a**) the Ni(111) and (**c**) sapphire. (Insets) Corresponding FFT patterns of each image. Cross-sectional HR-TEM images of the h-BN films on (**b**) the Ni(111) and (**d**) sapphire.

Microstructural and chemical properties of the h-BN films grown on the Ni(111) and on sapphire were characterized by HR-TEM, NEXAFS spectroscopy, and XPS. Figure 3 shows plan-view and cross-sectional HR-TEM micrographs of the h-BN films grown on the Ni(111) and on sapphire. The fast Fourier transform (FFT) pattern of the h-BN film grown on the Ni(111) (inset of Fig. 3a) exhibits a clear set of 6-fold symmetric spots, indicating that the h-BN domains constituting the film have crystallographic homogeneity. Cross-sectional HR-TEM image of the h-BN film on the Ni(111) shows a clearly layered structure of 6–7 layers (Fig. 3b). On the other hand, this shows a large contrast to the h-BN film grown on sapphire which shows a more defective layered structure (Fig. 3c,d). The FFT pattern of the h-BN film grown on sapphire (inset of Fig. 3c) exhibits a 6-fold symmetry with diffused spots as a result of grain misalignment and crystalline imperfections in the h-BN film. See additional cross-sectional HR-TEM images in Supplementary Fig. S10.

**Figure 4:**
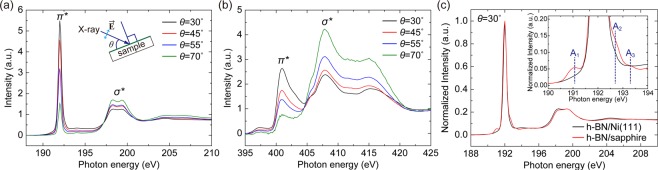
NEXAFS spectroscopy. (**a**) B K-edge spectra and (**b**) N K-edge spectra of the h-BN film grown on the Ni(111) for different incident angles *θ* (the angle between the sample plane and the X-ray propagating direction, as marked in the figure). (**c**) B K-edge spectra of the h-BN films grown on the Ni(111) and sapphire, measured at the incident angle *θ* of 30°. (Inset) Magnified spectra in the range between 190 eV and 194 eV for showing the low-energy shoulder A_1_, the high-energy shoulders A_2_, A_3_.

The structural properties of h-BN were further investigated by NEXAFS analyses. Figure 4a,b shows B K-edge and N K-edge NEXAFS spectra acquired from the h-BN film grown on the Ni(111), respectively, for different X-ray incident angle *θ* between the sample plane and the X-ray propagating direction. The NEXAFS peaks at 192.0 eV in the B K-edge spectra (Fig. 4a) and at 401.0 eV in the N K-edge spectra (Fig. 4b) originate from the transitions of core-level electrons into the π* states of h-BN^37–39^. On the other hand, the high energy features of both B K-edge and N K-edge NEXAFS spectra come from the transitions of core-level electrons into the σ* states of h-BN^37–39^. These spectra reveal that B and N atoms are sp^2^-hybridized, implying the hexagonal structure of the grown BN film. In addition, as the incident angle *θ* increases from 30° to 70°, the intensity of π* peaks decrease while the σ* peaks increase in both NEXAFS spectra, indicating that each h-BN atomic layer on the Ni(111) is nearly parallel to each other and to the surface of the substrate^37^. Similarly, the h-BN film on sapphire shows spectral features reflecting electronic transitions into π* and σ* states, which indicates the sp^2^-hybridized structure^37^ (see Supplementary Fig. S11). Figure 4c compares the normalized B K-edge spectra from the h-BN films grown on the Ni(111) and on sapphire, taken at the X-ray incident angle of 30°. The spectra are almost identical, except for distinctive shoulders of the π* peak appearing in the h-BN film on sapphire (the inset of Fig. 4c). The low-energy shoulder A_1_ at around 191.0 eV represents metallic-like B-B bonds from boron clusters^40^. On the other hand, the high-energy shoulders A_2_ near 192.7 eV and A_3_ at around 193.3 eV represent B bonding with two N and one O atoms, and B bonding with one N and two O atoms, respectively^40^. In other words, nitrogen vacancies are generated during the growth, leaving behind boron dangling bonds. The energetically unstable boron dangling bonds are readily saturated by oxygen atoms when exposed to air. This indicates that the h-BN film grown on sapphire contains a much larger amount of atomic disorders such as boron clusters and nitrogen vacancies compared to the h-BN film on the Ni(111). In addition, XPS chemical analyses reveal the h-BN film on sapphire has a higher B/N ratio and an additional bonding state that is attributed to the BN_x_O_y_ component, implying the more defective nature of the h-BN film on sapphire (see Supplementary Note 3).

With the experimental observations, we turn to discuss the mechanism involved in the growth of h-BN relying on the catalytic influence of the underlying substrates. Because the growth temperature is far higher than the decomposition temperature of TEB^41^, dehydrogenated TEB is adsorbed on the surface as B atoms or B-containing radicals regardless of the substrate. Therefore, the adsorption and decomposition of NH_3_ on the surface of the substrate is very likely to be the key steps determining the growth behavior of h-BN. A high thermal dissociation energy is required for hydrogen dissociation of NH_3_ (NH_3_ → NH_2_ + H, 4.8 eV)^42^, which may result in marginal NH_3_ decomposition under the given growth temperature unless some kind of catalysis is in effect. Using density functional theory (DFT) calculations, X. Duan *et al*. showed the adsorption and decomposition of NH_3_ and its radicals can be catalytically facilitated on the Ni(111) surface, where the hydrogen dissociation reaction of NH_3_ encounters a significantly lower energy barrier of 1.11 eV^43^. The elemental B and N atoms released from the decomposition of TEB and NH_3_ precursors are adsorbed on the Ni(111) surface, as the first step towards the formation of h-BN lattice. The theoretical investigation by S. Liu *et al*.^44^ suggests that the adsorbed B and N atoms on the Ni(111) surface initiate the formation of linear BN structures, which subsequently evolve into branched and then hexagonal structures serving as the nucleus for further h-BN growth. The formation of a continuous h-BN sheet on the Ni(111) substrate is energetically favored at elevated growth temperatures in the region of 1300 K^44^. On the other hand, in the absence of catalytic activity, most NH_3_ molecules remain intact at the substrate surface owing to the very low degree of NH_3_ decomposition at typical MOCVD growth temperatures^45^, which causes a significant reduction in activated V/III ratio. The incomplete decomposition of NH_3_ precursor can be the bottleneck hampering the formation of h-BN layers on sapphire due to the lack of available N adatoms. As a result, the h-BN film on sapphire grown at the relatively low temperature of 1050 °C has substantial atomic disorders including boron clusters, nitrogen vacancies, and disordered bonding states as shown in the Raman spectroscopy, HR-TEM, NEXAFS spectroscopy, and XPS studies.

**Figure 5:**
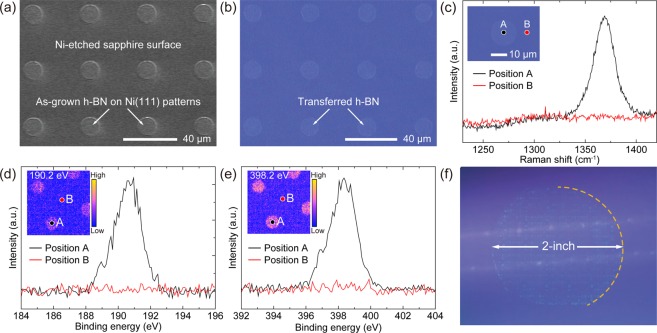
Selective-area growth of h-BN. (**a**) SEM image of as-grown h-BN on the pre-patterned Ni(111) template and (**b**) corresponding optical image after transfer onto SiO_2_/Si. (**c**) Raman spectra acquired from the positions marked in the inset optical image. Micro-focused XPS spectra of (**d**) B 1 s and (**e**) N 1 s core-levels measured at specific positions marked in the inset images. (Inset) SPEM images for selected energies of 190.2 eV and 398.2 eV over 80 × 80 μm^2^ scan range, respectively. (**f**) Photograph of the wafer-scale selective-area grown h-BN after transfer onto a 4-inch SiO_2_/Si substrate.

Based on the experimental results, selective-area growth of h-BN at desired locations with micro-scale, and even nano-scale, patterned geometries can be enabled by taking advantage of different catalytic activities of underlying substrates. Figure 5 demonstrates the micro-scale selective-area growth of h-BN using a pre-patterned Ni(111) template on sapphire. A cubic array of circles with a diameter of 17 μm and pitch of 48 μm was defined on the 2-inch Ni(111) template by conventional photolithography, followed by wet etching of Ni, forming the array of Ni pillars on sapphire. The selective-area growth of h-BN was performed on the Ni patterns covering the whole 2-inch sapphire with the same condition used for the growth on the Ni(111) template. Figure 5a is a top-view SEM image of as-grown h-BN on the pre-patterned Ni(111) template and Fig. 5b is a corresponding optical image after transferring the grown h-BN layers onto a SiO_2_/Si substrate. Due to the predominant catalytic effect of the Ni(111) patterned area in comparison with the Ni-etched sapphire, a large difference in both growth rate and crystal quality results in the high quality h-BN layers being selectively grown on the Ni(111) patterns. To ensure that the h-BN growth is spatially confined on the Ni(111) patterned area, Raman spectroscopy and scanning photoelectron microscopy (SPEM) were carried out. As shown in Fig. 5c, there is a characteristic Raman peak measured from the transferred h-BN layers on the Ni(111) patterned area, while no such a peak is observed at the Ni-etched sapphire surface, indicating the localized growth of h-BN on Ni(111). The local chemical states of the selective-area grown h-BN layers were investigated using SPEM. Figure 5d,e shows spatially resolved XPS spectra and SPEM images acquired for the B 1 s and the N 1 s core-level energies. The characteristic B 1 s and N 1 s photoelectron spectra are only observed on the h-BN domains, which is consistent with the XPS results of h-BN on the Ni(111) template (see Supplementary Fig. S3 and Supplementary Table S1). The slight blue-shift and the broadening of the peaks are attributed to the surface charge buildup on the spatially-isolated and insulating h-BN^46^. Additional SPEM images for B 1 s and N 1 s photoelectrons acquired at various energy windows are shown in Supplementary Fig. S12. The SPEM measurement confirms the spatially confined growth of h-BN on the Ni(111) patterned area. Figure 5f is a photograph of wafer-scale selective-area grown h-BN after transfer onto a 4-inch SiO_2_/Si substrate. This technique for the selective-area growth of h-BN on a wafer level can significantly contribute to the capabilities of h-BN as a fundamental building block for large-scale 2D materials-based device fabrication and integration.

## Conclusions

In summary, we have demonstrated the wafer-scale growth of high-quality few-layer h-BN film on Ni(111) template at the relatively low temperature of 1000 °C using a MOCVD system. It is shown that the catalytic effect of the substrate is a critical factor determining the growth kinetics as well as the crystal quality of h-BN. The Ni(111) template can facilitate the adsorption and decomposition of NH_3_ and its radicals, leading to the formation of high-quality h-BN film. In contrast, the NH_3_ precursor is incompletely decomposed on the inert sapphire, resulting in the formation of low-crystalline h-BN film at a slower growth rate. In addition, we have demonstrated selective-area growth of h-BN at desired locations with micro-scale geometries by using a photo-lithographically pre-patterned Ni(111) template on sapphire. We believe that these findings have advanced our understanding of the growth mechanism of h-BN by MOCVD and will contribute an important step toward scalable and controllable production of high-quality h-BN building blocks for a variety of practical applications.

## Methods

### Preparation of Ni(111) template on sapphire

A Ni film (~600 nm thickness) was deposited on a *c*-plane sapphire substrate *via* sputtering of a polycrystalline Ni target. The sputtering chamber was vacuumed to a based pressure of 7 × 10^−7^ Torr, then filled with Ar gas to a working pressure of 7.0 mTorr. The Ni film was deposited with a sputtering power of 50 W. The as-deposited Ni template was then loaded to a multi-wafer (11 × 2-inch wafers) MOCVD reactor (AIXTRON, AIX 2400 G3-HT, Supplementary Fig. S13) and annealed in H_2_ atmosphere at 1050 °C, 150 mbar for 20 min to obtain clean and smooth Ni(111) template. Note that the thickness optimization of the Ni film is discussed in Supplementary Note 4.

### MOCVD growth of h-BN films

After the annealing process, an h-BN film was grown on the Ni(111) template at 1000 °C, 30 mbar in the MOCVD reactor. A pulsed-mode growth with alternating supplies of triethylborane (TEB) and ammonia (NH_3_) using H_2_ carrier gas was applied to restrain gas phase pre-reactions. The procedure of the pulsed-mode growth was composed of 4 steps per period: (1) 5 seconds of TEB injection, (2) 2 seconds of H_2_ interruption, (3) 4 seconds of NH_3_ injection, and (4) 2 seconds of H_2_ interruption. The flow rates of TEB and NH_3_ were maintained at 10 sccm and 8000 sccm, respectively, corresponding to the V/III ratio of around 23500 with consideration for the individual source injection times. A total of 50 source injection periods were applied for the growth of h-BN films on Ni(111). As a control experiment, the h-BN film was also grown on a bare *c*-plane sapphire substrate at 1050 °C, 30 mbar for 200 periods due to the slower growth rate.

### Selective-area growth of h-BN

Conventional photolithography was used to fabricate a pre-patterned Ni(111) template on sapphire for selective-area growth of h-BN. First, a photoresist was spin-coated on the Ni template on sapphire, followed by a 100 °C baking on a hot plate. After pattern transfer and development, FeCl_3_ reagent was used to etch the patterned Ni template. The sample was then cleaned using solvents such as acetone, isopropyl alcohol (IPA), and distilled water, leaving a well-defined array of Ni pillars. Afterwards, the pre-patterned Ni template was loaded into the MOCVD reactor and annealed in H_2_ atmosphere at 1050 °C, 150 mbar for 20 min. Finally, h-BN was grown at 1000 °C, 30 mbar for 50 source injection periods.

### Transfer method

As-grown h-BN samples were spin-coated with poly(methyl methacrylate) (PMMA) as a supportive layer. The PMMA/h-BN films grown on Ni(111) were immersed into FeCl_3_ reagent, while those on sapphire were etched in diluted hydrofluoric acid. Subsequently, the PMMA/h-BN films were delaminated from the underlying substrates and washed by distilled water to remove contaminations. Then, the PMMA/h-BN films were transferred onto the target substrates. After drying the samples, the PMMA layer was dissolved with acetone to yield the h-BN films for subsequent characterizations.

### Characterizations

Surface morphology and structural properties of the Ni film was measured by using AFM (Veeco, Dimension 3100, tapping mode), XRD (Bruker D8 HRXRD with Cu K-α radiation), and SEM (Philips electron optics B.V., XL 30 S FEG) equipped with EBSD (EDAX Japan K.K, OIM 4000). Surface morphology of the h-BN film was also observed by using SEM (Philips electron optics B.V., XL 30 S FEG). Optical absorbance spectra of the h-BN films transferred onto a double-side-polished sapphire wafer were obtained by using UV−vis−NIR spectrometer (Perkin Elmer, Lambda 750 S) at room temperature. Raman spectroscopy measurements were carried out by using a 532 nm Nd:YaG laser and a charge-coupled device (CCD) camera operated at −60 °C. Plan-view HR-TEM analysis was exploited for the h-BN films transferred on TEM grids by using a JEOL JEM-ARM 200 F with a Cs-corrected probe operated at 60 kV. Cross-sectional HR-TEM characterization was performed by using a JEOL 2100 F microscope operated at 80 kV. An in-house XPS instrument with an Al K-α radiation source was employed to estimate the stoichiometry of the h-BN film on sapphire. High-resolution NEXAFS and XPS measurements were carried out using synchrotron X-ray radiation at the 4D and 10D beamlines of Pohang Accelerator Laboratory (PAL) in Korea, respectively. The SPEM measurement for the selective-area grown h-BN after transfer onto a SiO_2_/Si substrate was carried out at the 8A1 beamline of PAL. The photon energy was set to 705 eV and the incident X-ray was focused to be approximately 200 nm × 200 nm in size with the use of a Fresnel zone plate. The mapping images of photoelectron intensity were simultaneously obtained at various windows of binding energy. The width of binding energy window was 1.0 eV.

## Supplementary information

Supplementary information

## Data Availability

The datasets generated and/or analyzed during the current study are available from the corresponding author on reasonable request.

## References

[1] Pakdel A, Bando Y, Golberg D (2014). Nano boron nitride flatland. Chem. Soc. Rev..

[2] Lipp A, Schwetz KA, Hunold K (1989). Hexagonal boron nitride: Fabrication, properties and applications. J. Eur. Ceram. Soc..

[3] Gupta A, Sakthivel T, Seal S (2015). Recent development in 2D materials beyond graphene. Prog. Mater. Sci..

[4] Song L (2010). Large scale growth and characterization of atomic hexagonal boron nitride layers. Nano Lett..

[5] Kim KK (2012). Synthesis of monolayer hexagonal boron nitride on Cu foil using chemical vapor deposition. Nano Lett..

[6] Xu M, Liang T, Shi M, Chen H (2013). Graphene-like two-dimensional materials. Chem. Rev..

[7] Dean CR (2010). Boron nitride substrates for high-quality graphene electronics. Nat. Nanotechnol..

[8] Mayorov AS (2011). Micrometer-scale ballistic transport in encapsulated graphene at room temperature. Nano Lett..

[9] Britnell L (2012). Electron tunneling through ultrathin boron nitride crystalline barriers. Nano Lett..

[10] Kubota Y, Watanabe K, Tsuda O, Taniguchi T (2007). Deep ultraviolet light-emitting hexagonal boron nitride synthesized at atmospheric pressure. Science.

[11] Watanabe K, Taniguchi T, Kanda H (2004). Direct-bandgap properties and evidence for ultraviolet lasing of hexagonal boron nitride single crystal. Nat. Mater..

[12] Shi Y (2010). Synthesis of few-layer hexagonal boron nitride thin film by chemical vapor deposition. Nano Lett..

[13] Oh H (2016). Centimeter-sized epitaxial h-BN films. NPG Asia Mater..

[14] Lee KH (2012). Large-scale synthesis of high-quality hexagonal boron nitride nanosheets for large-area graphene electronics. Nano Lett..

[15] Park J-H (2014). Large-area monolayer hexagonal boron nitride on Pt foil. ACS Nano.

[16] Kobayashi K, Kumakura K, Akasaka T, Makimoto T (2012). Layered boron nitride as a release layer for mechanical transfer of GaN-based devices. Nature.

[17] Kobayashi K, Akasaka T (2008). Hexagonal BN epitaxial growth on (0001) sapphire substrate by MOVPE. J. Cryst. Growth.

[18] Paduano QS, Snure M, Bondy J, Zens TWC (2014). Self-terminating growth in hexagonal boron nitride by metal organic chemical vapor deposition. Appl. Phys. Express.

[19] Paduano Q (2016). Metalorganic chemical vapor deposition of few-layer sp^2^ bonded boron nitride film. J. Cryst. Growth.

[20] Kim DY (2017). Pressure-dependent growth of wafer-scale few-layer h-BN by metal-organic chemical vapor deposition. Cryst. Growth Des..

[21] Kim DY (2017). Role of hydrogen carrier gas on the growth of few layer hexagonal boron nitrides by metal-organic chemical vapor deposition. AIP Adv..

[22] Nakamura K (1986). Preparation and properties of boron nitride films by metal organic chemical vapor deposition. J. Electrochem. Soc..

[23] Rice A (2018). Effects of deposition temperature and ammonia flow on metal-organic chemical vapor deposition of hexagonal boron nitride. J. Cryst. Growth.

[24] Jang A-R (2016). Wafer-scale and wrinkle-free epitaxial growth of single-orientated multilayer hexagonal boron nitride on sapphire. Nano Lett..

[25] Kobayashi Y, Akasaka T, Makimoto T (2008). Hexagonal boron nitride grown by MOVPE. J. Cryst. Growth.

[26] Ding D, Solis-Fernandez P, Hibino H, Ago H (2016). Spatially controlled nucleation of single-crystal graphene on Cu assisted by stacked Ni. ACS Nano.

[27] Kim H-J, Kim H, Yang S, Kwon J-Y (2017). Grains in selectively grown MoS_2_ thin films. Small.

[28] Thiele S (2010). Engineering polycrystalline Ni films to improve thickness uniformity of the chemical-vapor-deposition-grown graphene films. Nanotechnology.

[29] Carel R, Thompson CV, Frost HJ (1996). Computer simulation of strain energy effects vs surface and interface energy effects on grain growth in thin films. Acta Mater..

[30] Chugh D (2018). Flow modulation epitaxy of hexagonal boron nitride. 2D Mater..

[31] Jiang HX, Lin JY (2017). Review—Hexagonal boron nitride epilayers: growth, optical properties and device applications. ECS J. Solid State Sci. Technol..

[32] Deng B (2017). Wrinkle-free single-crystal graphene wafer grown on strain-engineered substrates. ACS Nano.

[33] Preobrajenski AB, Vinogradov AS, Mårtensson N (2004). Ni 3d–BN π hybridization at the h-BN/Ni(111) interface observed with core-level spectroscopies. Phys. Rev. B.

[34] Geick R, Perry CH, Rupprecht G (1966). Normal modes in hexagonal boron nitride. Phys. Rev..

[35] Gorbachev RV (2011). Hunting for monolayer boron nitride: Optical and Raman signatures. Small.

[36] Ismach A (2012). Toward the controlled synthesis of hexagonal boron nitride films. ACS Nano.

[37] Jiménez I (1997). Core-level photoabsorption study of defects and metastable bonding configurations in boron nitride. Phys. Rev. B: Condens. Matter Mater. Phys..

[38] Laskowski R, Gallauner T, Blaha P, Schwarz K (2009). Density functional theory simulations of B K and N K NEXAFS spectra of h-BN/transition metal(111) interfaces. J. Phys.: Condens. Matter.

[39] Tonkikh AA (2016). Structural and electronic properties of epitaxial multilayer h-BN on Ni(111) for spintronics Applications. Sci. Rep..

[40] Petravic M (2010). Decoration of nitrogen vacancies by oxygen atoms in boron nitride nanotubes. Phys. Chem. Chem. Phys..

[41] Lewis JS (1996). Chemical vapor deposition of boron-carbon thin films using organometallic reagents. Mater. Lett..

[42] Konuma, M. *Film deposition by plasma techniques Ch. 7* (Springer-Verlag, Berlin, 1992).

[43] Duan XZ (2012). Ammonia decomposition on Fe(110), Co(111) and Ni(111) surfaces: A density functional theory study. J. Mol. Catal. A: Chem..

[44] Liu S, van Duin AC, van Duin DM, Liu B, Edgar JH (2017). Atomistic insights into nucleation and formation of hexagonal boron nitride on nickel from first-principles-based reactive molecular dynamics simulations. ACS Nano.

[45] Liu SS, Stevenson DA (1978). Growth kinetics and catalytic effects in the vapor phase epitaxy of gallium nitride. J. Electrochem. Soc..

[46] Cros A (1992). Charging effects in X-ray photoelectron spectroscopy. J. Electron Spectrosc. Relat. Phenom..

