# Supplementary material for: Wafer-scale and selective-area growth of high-quality hexagonal boron nitride on Ni(111) by metal-organic chemical vapor deposition
Source: Sci Rep. 2019 Apr 5;9:5736. doi: 10.1038/s41598-019-42236-4 (PMC6450880; doi:10.1038/s41598-019-42236-4)
Supplement: Supplementary file 1 — Supplementary information [file 41598_2019_42236_MOESM1_ESM.docx]

**Supplementary Information**

Wafer-scale and selective-area growth of high-quality hexagonal boron nitride on Ni(111) by metal-organic chemical vapor deposition

Hokyeong Jeong^1^, Dong Yeong Kim^1^, Jaewon Kim^1^, Seokho Moon^1^, Nam Han^1^, Seung Hee Lee^1^, Odongo Francis Ngome Okello^1^, Kyung Song^2^, Si-Young Choi^1,2^, and Jong Kyu Kim^1,*^

^1^Department of Materials Science and Engineering, Pohang University of Science and Technology (POSTECH), Pohang 37673, Republic of Korea

^2^Materials Modeling and Characterization Department, Korea Institute of Materials Science (KIMS), Changwon 51508, Republic of Korea

^*^Correspondence: Jong Kyu Kim ([kimjk@postech.ac.kr](mailto:kimjk@postech.ac.kr))

**Supplementary Figures**


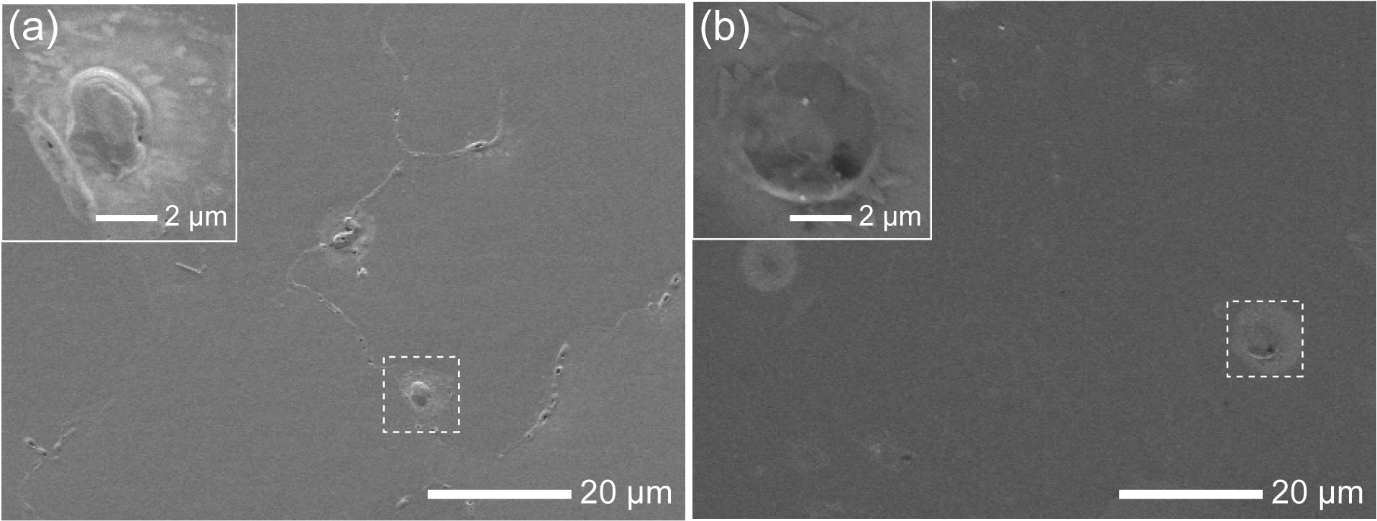


**Supplementary Figure S1.** SEM images of (**a**) as-grown h-BN film on the Ni(111) template and (**b**) the transferred h-BN film. (Insets) Higher-magnification images of the marked regions, exhibiting the irregular formation of triangular h-BN domains.


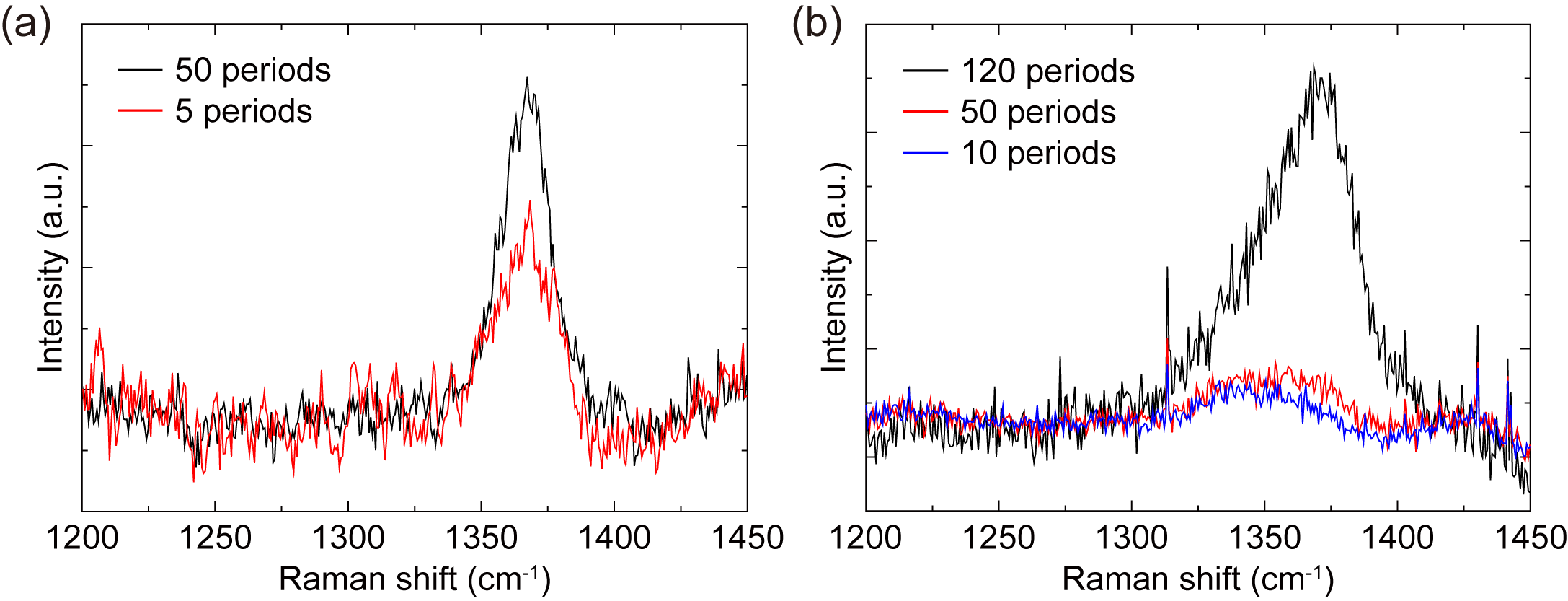


**Supplementary Figure S2.** Raman spectroscopy measurements. Raman spectra of h-BN grown on (**a**) the Ni(111) and (**b**) sapphire for different source injection periods.


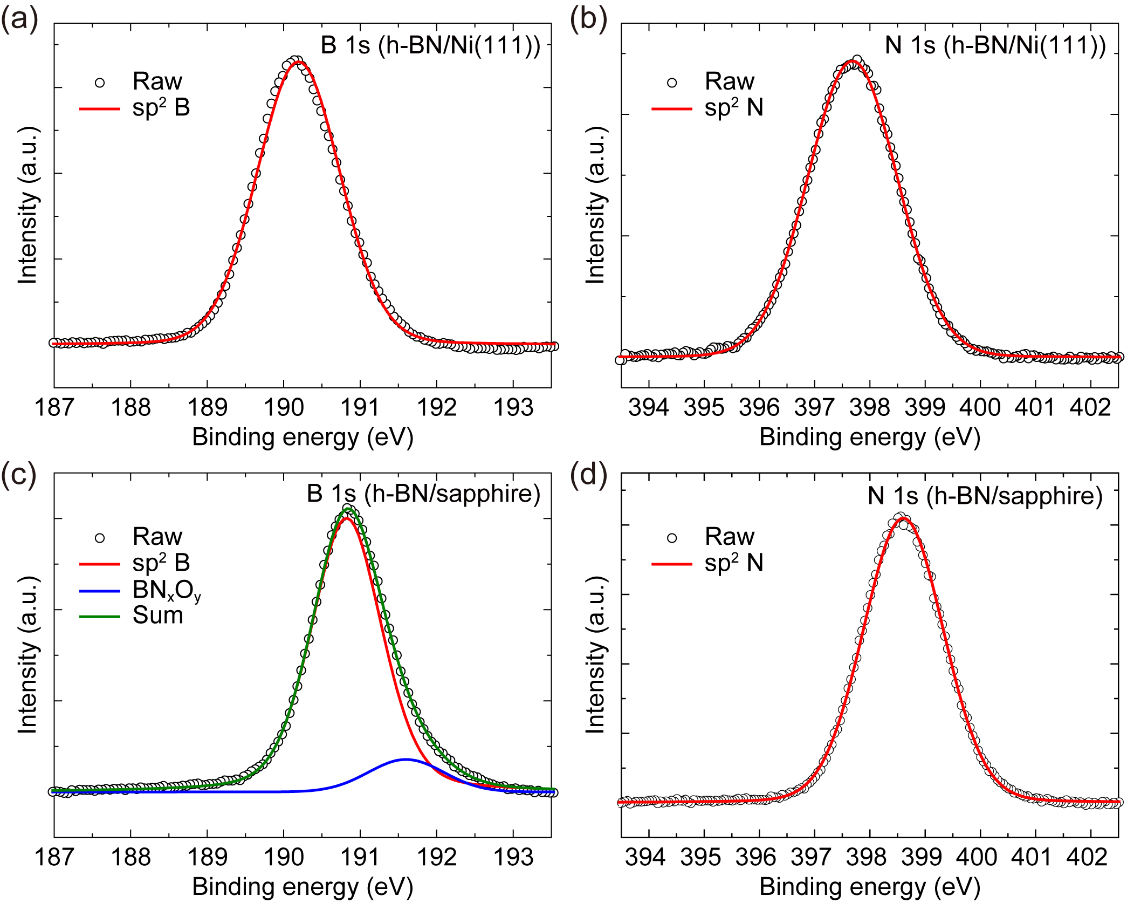


**Supplementary Figure S3.** Synchrotron-radiation-based high-resolution XPS analysis. (**a**) B 1s and (**b**) N 1s core-level spectra from the h-BN film on the Ni(111). (**c**) B 1s and (**d**) N 1s core-level spectra from the h-BN film on sapphire.


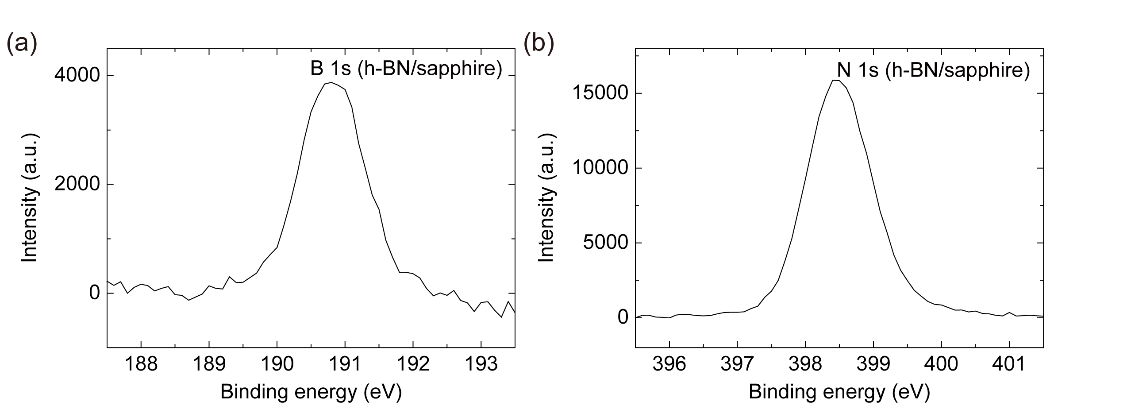


**Supplementary Figure S4.** In-house XPS measurement of the h-BN film on sapphire for stoichiometry analysis. (**a**) B 1s and (**b**) N 1s core-level spectra.


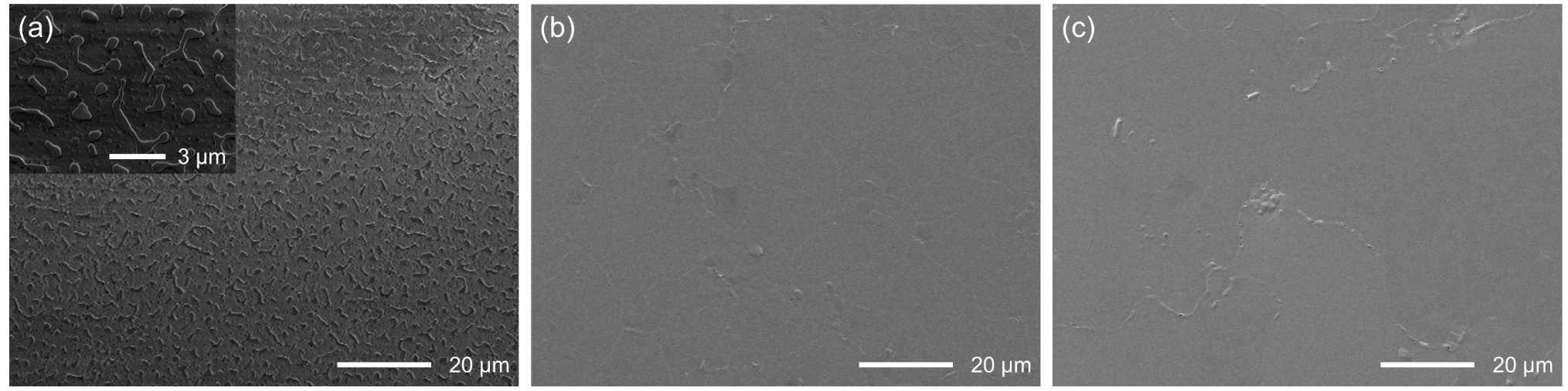


**Supplementary Figure S5.** SEM images of MOCVD-grown h-BN films on Ni films with different thicknesses of (**a**) 100 nm, (**b**) 300 nm, and (**c**) 600 nm.


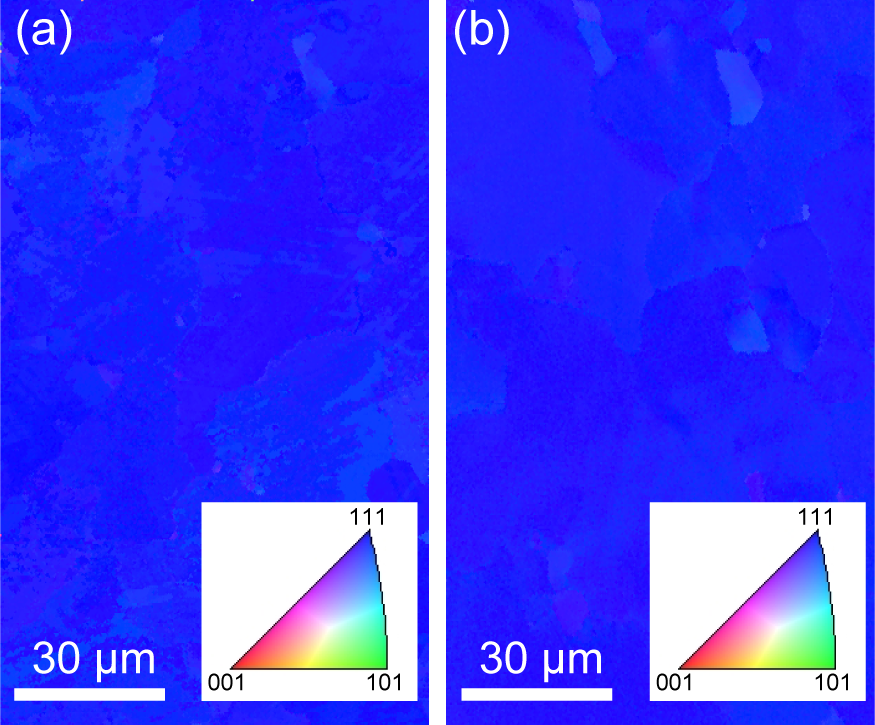


**Supplementary Figure S6.** EBSD crystallographic mapping images of annealed Ni films with a thickness of (**a**) 300 nm and (**b**) 600 nm. (Insets) Inverse pole figure color triangle for crystallographic orientations.


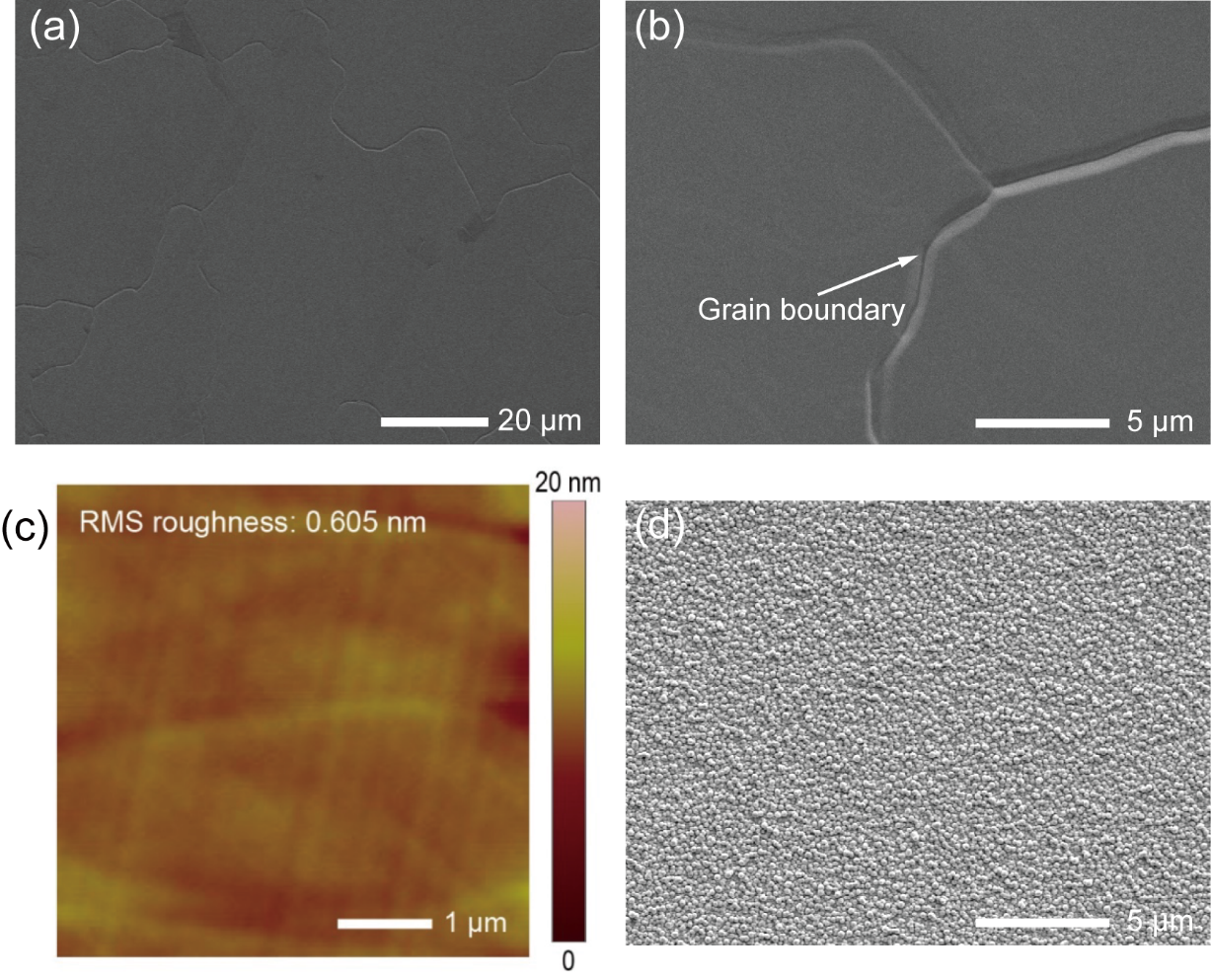


**Supplementary Figure S7.** Surface morphology of the Ni(111) template. (**a, b**) Top-view SEM images and (**c**) AFM image of the Ni(111) template after the thermal annealing, showing a smooth surface topography with a root-mean-square (RMS) roughness of 0.605 nm. (**d**) Top-view SEM image of as-deposited Ni template, exhibiting an uneven surface topography.


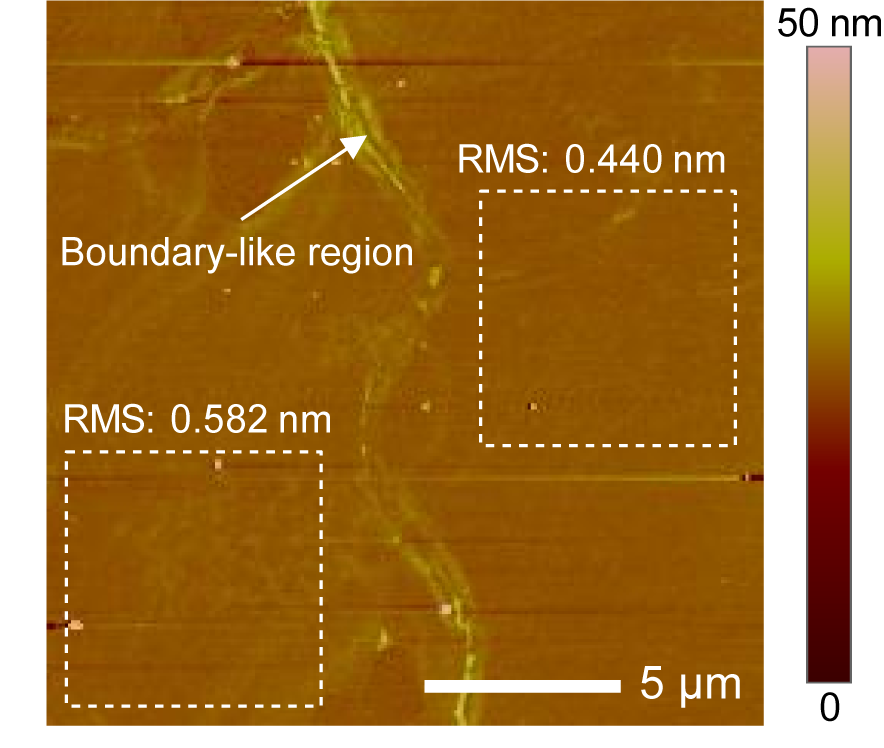


**Supplementary Figure S8.** AFM surface morphology of the h-BN film transferred on a SiO_2_/Si substrate, taken at the boundary-like region. The transferred h-BN film exhibits boundary-like regions that seem to originate from the grain boundaries of the annealed Ni(111) template. The wrinkle-free feature may result from the strong interfacial interaction between h-BN and Ni(111)^9,10^. h-BN is supposed to be strongly chemisorbed on the Ni(111) surface^9^, allowing strain energy retaining in the h-BN lattice instead of being released in the form of wrinkles^10^.


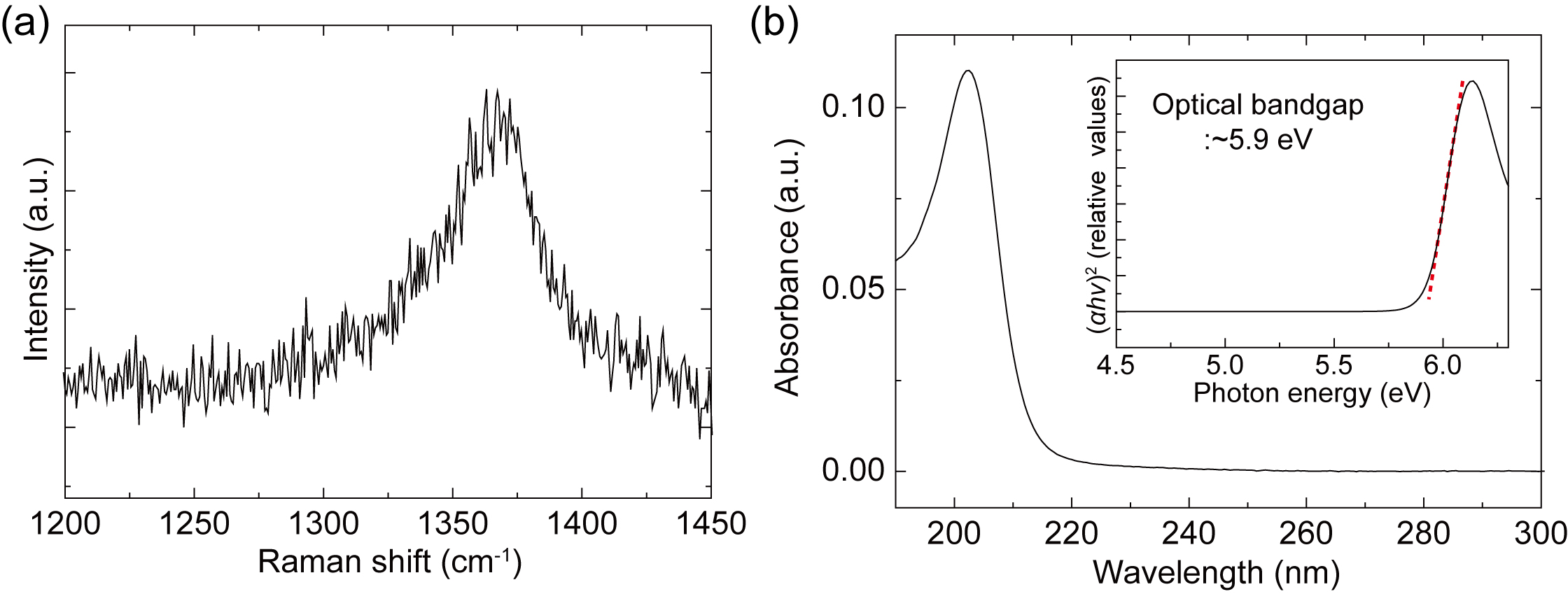


**Supplementary Figure S9.** Spectroscopic characterization of h-BN on sapphire. (**a**) Raman spectrum and (**b**) absorbance spectrum of the h-BN film grown on sapphire. (Inset) Tauc’s plot and optical band gap analysis.


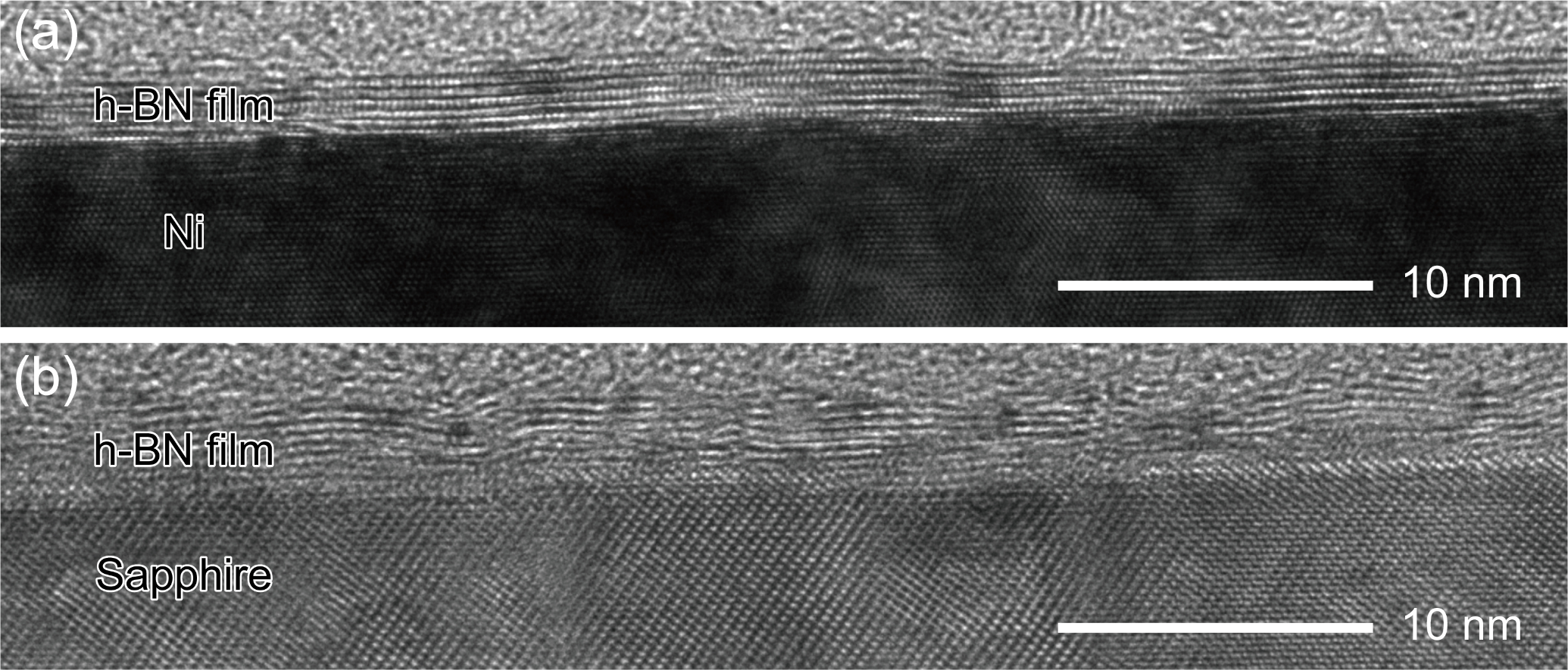


**Supplementary Figure S10.** Cross-sectional HR-TEM investigation. Cross-sectional HR-TEM images of the h-BN films grown on (**a**) the Ni(111) and (**b**) sapphire.


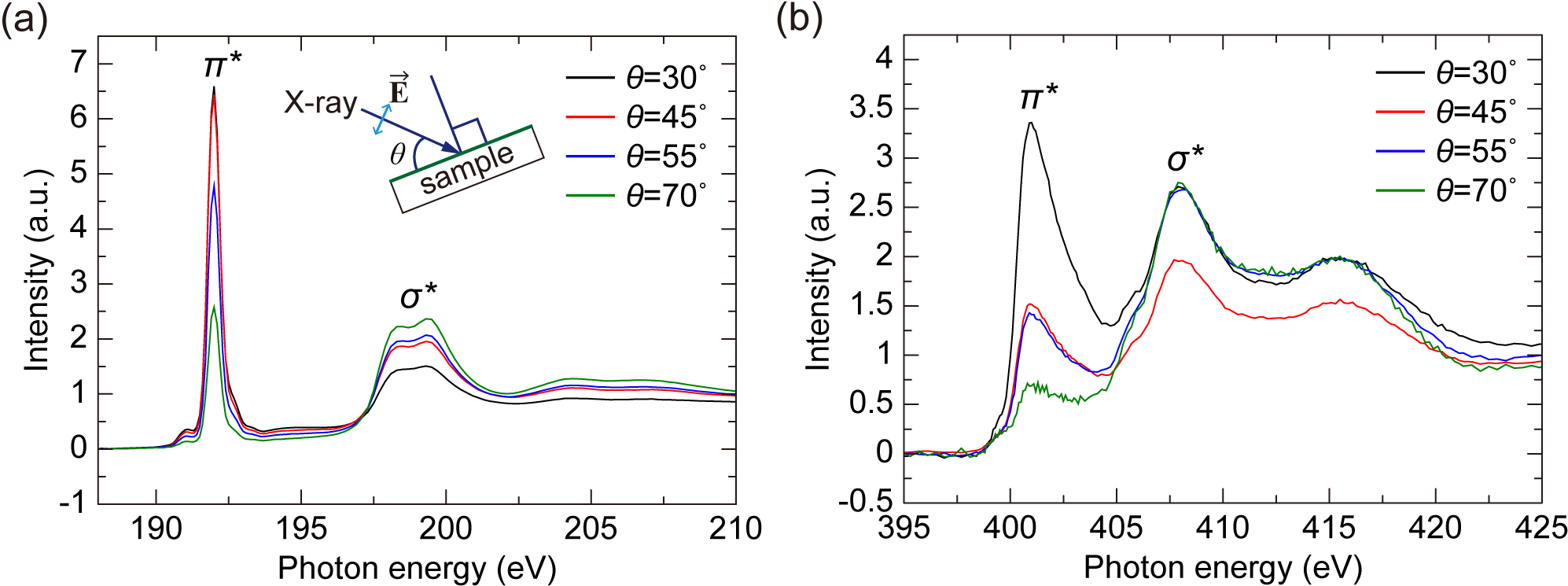


**Supplementary Figure S11.** NEXAFS spectra of the h-BN film grown on sapphire. (**a**) B K-edge spectra and (**b**) N K-edge spectra for different incident angle *θ* (the angle between the sample plane and the photon propagating direction, as marked in the figure).


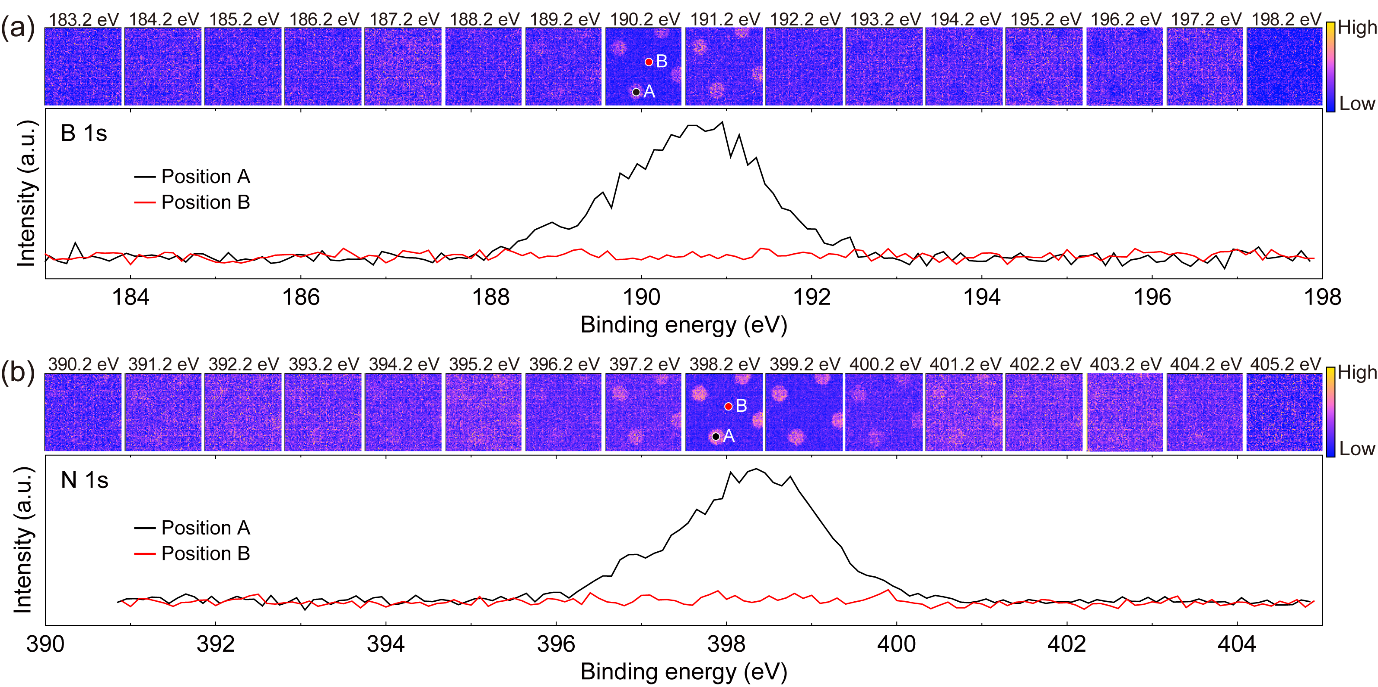


**Supplementary Figure S12.** SPEM characterization. (**a**) (Upper panel) SPEM images for B 1s core-level photoelectrons, simultaneously obtained at various energy windows over 80 × 80 μm^2^ scan range. (Bottom panel) Microfocused XPS spectra of B 1s core-level measured at the marked positions. (**b**) SPEM images for N 1s core-level photoelectrons, simultaneously acquired at various energy windows. Microfocused XPS spectra of N 1s core-level obtained at the same position above.


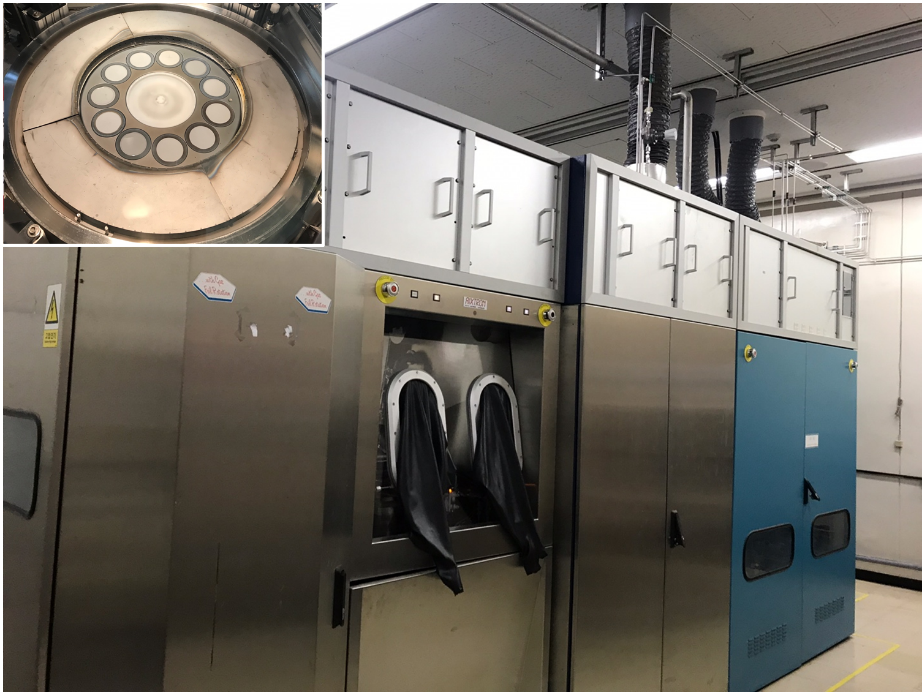


**Supplementary Figure S13.** Photograph of MOCVD system. A photograph of AIXTRON AIX G3-2400 HT MOCVD system used in this study. (Inset) Reactor chamber with capacity of 11×2-inch wafers.

**Supplementary Tables**

**Supplementary Table S1.** Summary of the fitted XPS results for the h-BN films on the Ni(111) and sapphire, shown in Supplementary Fig. S3.

|  | sp^2^ B | | BN_x_O_y_ | | sp^2^ N | |
| --- | --- | --- | --- | --- | --- | --- |
| Substrate | Binding energy (eV) | FWHM (eV) | Binding energy (eV) | FWHM (eV) | Binding energy (eV) | FWHM (eV) |
| Ni(111) | 190.2 | 1.28 | absent | absent | 397.7 | 1.91 |
| Sapphire | 190.8 | 1.10 | 191.6 | 1.16 | 398.6 | 1.72 |

**Supplementary Table S2.** Chemical analysis data of the h-BN film on sapphire, shown in Supplementary Fig. S4.

|  | Binding energy (eV) | Intensity (a.u.) | Area (eV) | Atomic % | B/N ratio |
| --- | --- | --- | --- | --- | --- |
| B 1s | 190.8 | 4104.4 | 5900.3 | 15.83% | 1.24 |
| N 1s | 398.5 | 15780.3 | 20168.4 | 12.75% |  |

**Supplementary Notes**

**Supplementary Note 1. Defective surface features of the h-BN film on the Ni(111).**

Supplementary Fig. 1a is a high magnification SEM image of the as-grown h-BN on the Ni(111) template. There are particle-like features on the surface that should be discussed. We believe the particle-like surface features can be attributed to hydrogen blistering of the Ni surface. When the Ni film is exposed to hydrogen under elevated temperatures, hydrogen atoms are able to diffuse into the Ni and form hydrogen blisters^1^. Interestingly, the hydrogen blisters were observed on the h-BN grown Ni surface, while being rarely observed on the Ni just annealed in hydrogen ambient. The formation of hydrogen blisters was probably enhanced by carbon impurities^2^ which were released from the decomposed TEB precursor during h-BN growth. As shown in Supplementary Fig. 1b, the irregular formation of thicker triangular h-BN domains were observed around the hydrogen blisters due to its defective stepped morphology.

**Supplementary Note 2. Comparison of h-BN growth rates between on the Ni(111) and sapphire.**

We compared the growth rates of h-BN on the Ni(111) and sapphire by measuring Raman spectroscopy. Supplementary Fig. S2a shows that the characteristic Raman E_2g_ peak is observed from h-BN grown on the Ni(111) for 5 source injection periods, indicating the formation of a h-BN film for a short growth time. On the other hand, the Raman peak is not observed from the h-BN growth on sapphire for 10 and 50 periods, for which the h-BN lattice can be not completely formed (Supplementary Fig. S2b). The broad Raman profile of h-BN is measured on sapphire after the 120 periods growth. This result indicates a slower growth rate of h-BN on sapphire than on the Ni(111).

**Supplementary Note 3. XPS characterization.**

Supplementary Fig. S3 is XPS spectra of B 1s and N 1s core-levels from the h-BN films grown on the Ni(111) and on sapphire, measured using a synchrotron-radiation-based high-resolution XPS instrument. The binding energies and FWHM values of the peaks are presented in Supplementary Table S1. For the h-BN film grown on the Ni(111), the highly symmetric B 1s and N 1s peaks are centered at 190.2 eV and 397.7 eV, indicating sp^2^-hybridized B and N atoms (Supplementary Fig. S3a,b)^3,4^. On the other hand, for the film grown on sapphire, the main peaks of B 1s and N 1s are observed at 190.8 eV and 398.6 eV, which are consistent with the previously reported values (Supplementary Fig. S3c,d)^5^. However, there is an additional B 1s bonding state at 191.6 eV which is attributed to the BN_x_O_y_ component^6^. The results from the NEXAFS and XPS analyses indicate that the h-BN film grown on sapphire has substantial atomic disorders including nitrogen vacancies, leaving behind boron dangling bonds. The energetically unstable boron dangling bonds are readily saturated by oxygen atoms when exposed to air^7^.

The B/N stoichiometry of the h-BN film on sapphire was estimated by employing an in-house XPS instrument with Al K-α radiation (Supplementary Fig. S4 and Supplementary Table S2). As summarized in Supplementary Table S2, the estimated B/N atomic ratio from the XPS chemical analysis is over 1, implying the deficiency of N atoms. The XPS measurements reveal that the h-BN film on sapphire has a higher B/N ratio and a disordered bonding state, indicating the more defective nature of the h-BN film on sapphire.

**Supplementary Note 4. Thickness optimization of the Ni film.**

To obtain a high-quality h-BN film, the epi-ready Ni substrate should retain a uniform smooth surface and high crystallinity. To optimize the quality of the Ni substrate, we investigated the growth of h-BN on Ni films prepared by sputter deposition with different thicknesses of 100, 300, and 600 nm. These substrates were thermal annealed in the MOCVD reactor at 1050 °C and 150 mbar for 20 min under H_2_ ambient, and the h-BN films were sequentially grown as described in the Methods section. Supplementary Fig. S5 shows top-view SEM images of MOCVD-grown h-BN films on the Ni substrates. It is observed that the 100 nm-thick Ni film became agglomerated upon thermal annealing and h-BN growth at the elevated temperatures. This is understandable as metallic thin films with a high surface to volume ratio are susceptible to agglomeration and clustering, which is a thermally activated process to decrease the interfacial energy between the metal film and substrate^8^. On the other hand, the surface morphologies of 300 and 600 nm-thick Ni films remained continuous upon the annealing and growth. We compared the grain size of the 300 and 600 nm-thick annealed Ni films using EBSD as shown in Supplementary Fig. S6. While both the Ni films have the (111) preferred orientation, the average grain size of the 300 nm-thick Ni film was estimated to be approximately 34 μm which is smaller than that of the thicker 600 nm Ni film (~ 75 μm). Due to the better crystal quality (larger grain size) and surface morphology, the 600 nm-thick Ni film was used for the growth of h-BN film.

REFERENCES

1. Ren, X. C. *et al*. A nucleation mechanism of hydrogen blister in metals and alloys. *Metall. Mater. Trans. A* **39**, 87−97 (2008).
2. Ueda, Y., Shimada, T. & Nishikawa, M. Impacts of carbon impurities in hydrogen plasmas on tungsten blistering. *Nucl. Fusion* **44**, 62−67 (2004).
3. Shi, Y. *et al.* Synthesis of few-layer hexagonal boron nitride thin film by chemical vapor deposition. *Nano Lett.* **10,** 4134–4139 (2010).
4. Ismach, A. *et al*. Toward the controlled synthesis of hexagonal boron nitride films. *ACS Nano* **6,** 6378–6385 (2012).
5. Jang, A.-R. *et al*. Wafer-scale and wrinkle-free epitaxial growth of single-orientated multilayer hexagonal boron nitride on sapphire. *Nano Lett.* **16,** 3360–3366 (2016).
6. Guimon, C. *et al*. XPS study of BN thin films deposited by CVD on SiC plane substrates. *Surf. Interface Anal.* **16**, 440–445 (1990).
7. Petravic, M. *et al*. Decoration of nitrogen vacancies by oxygen atoms in boron nitride nanotubes. *Phys. Chem. Chem. Phys.* **12**, 15349–15353 (2010).
8. Gadkari, P. R., Warren, A. P., Todi, R. M., Petrova, R. V. & Coffey, K. R. Comparison of the agglomeration behavior of thin metallic films on SiO_2_. *J. Vac. Sci. Technol. A* **23**, 1152 (2005).
9. Preobrajenski, A. B., Vinogradov, A. S. & Mårtensson, N. Ni 3d–BN π hybridization at the h-BN/Ni(111) interface observed with core-level spectroscopies. *Phys. Rev. B* **70**, 165404 (2004).
10. Deng, B. *et al*. Wrinkle-free single-crystal graphene wafer grown on strain-engineered substrates. *ACS Nano* **11**, 12337–12345 (2017).
